# Titin N2A Domain and Its Interactions at the Sarcomere

**DOI:** 10.3390/ijms22147563

**Published:** 2021-07-15

**Authors:** Adeleye O. Adewale, Young-Hoon Ahn

**Affiliations:** Department of Chemistry, Wayne State University, Detroit, MI 48202, USA; adeleye.adewale@wayne.edu

**Keywords:** titin, N2A domain, protein–protein interaction

## Abstract

Titin is a giant protein in the sarcomere that plays an essential role in muscle contraction with actin and myosin filaments. However, its utility goes beyond mechanical functions, extending to versatile and complex roles in sarcomere organization and maintenance, passive force, mechanosensing, and signaling. Titin’s multiple functions are in part attributed to its large size and modular structures that interact with a myriad of protein partners. Among titin’s domains, the N2A element is one of titin’s unique segments that contributes to titin’s functions in compliance, contraction, structural stability, and signaling via protein–protein interactions with actin filament, chaperones, stress-sensing proteins, and proteases. Considering the significance of N2A, this review highlights structural conformations of N2A, its predisposition for protein–protein interactions, and its multiple interacting protein partners that allow the modulation of titin’s biological effects. Lastly, the nature of N2A for interactions with chaperones and proteases is included, presenting it as an important node that impacts titin’s structural and functional integrity.

## 1. Introduction

**Figure 1:**
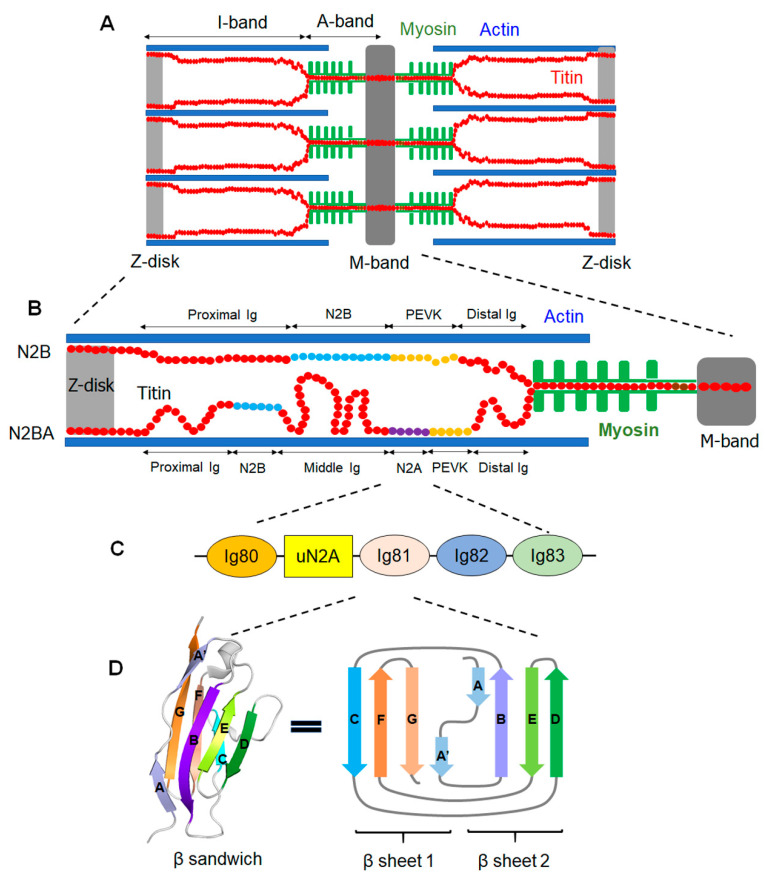
Structural organization of sarcomere, titin, and the N2A insertion of titin. (**A**) General depiction of the sarcomere in striated muscle showing the Z-disk demarcation of each sarcomere and the M-band in the middle of the sarcomere. The I-band and A-band sections of the sarcomere are indicated. The myofilament proteins, myosin (thick filament, green), actin (thin filament, blue), and titin (red), are shown. (**B**) Schematic representation of titin in striated cardiac muscle with a depiction of the N2B and N2BA isoforms in the half sarcomere. The N2BA isoform has additional segments, namely the middle immunoglobulins (Ig) and the N2A insertion. (**C**) Structural domains of the N2A insertion of titin. The N2A insertion has five sub-domains, among which four are immunoglobulin-like (Ig80, Ig81, Ig82, and Ig83) and one is a unique sequence (uN2A). (**D**) Crystal structure of the Ig81 sub-domain of N2A. The β-strands are aligned in an antiparallel orientation to form two groups of β-sheets, ABED and A’GFC. The β-sheets lie over each other in parallel to form a β-sandwich structure. (**A**,**B**) were adapted and redrawn from Koser et al.’s publication [4], and (**D**) was adapted and redrawn from Meyer and Wright’s publication [17].

The complexity of striated muscle is defined by the intricate organization of its components [1]. The involuntary cardiac and voluntary skeletal muscles are the primary types of striated muscle and are bundled as myofibers that consist of myofibrils arranged in parallel [2]. Myofibrils are composed of extended tandem repeats of sarcomeres that are the functional unit of muscle [3]. Importantly, the sarcomere encompasses multiple proteins within the sarcoplasm and extra-sarcoplasm that are essential for its structural framework and contractility [3]. Specifically, each sarcomere is defined within the borders of two Z-disks that consist of thin (actin) filament, thick (myosin) filament, and the giant protein titin, referred to as the third filament (Figure 1A) [2,4]. The actin and myosin myofilaments are directly involved in muscle contraction under the influence of an action potential, with consumption of the body’s energy currency, adenosine triphosphate (ATP) [2].

Titin spans the length of a half sarcomere, extending from its N-terminus in the Z-disk and terminating with its C-terminus in the M-line (Figure 1B), often described as a ‘molecular ruler’ that defines the length of the sarcomere [5,6]. Functionally, this giant protein has been reported to serve as a framework for sarcomerogenesis [5,7], maintenance of sarcomere length [8], alignment of the thick filament [9], and the maintenance of passive force and elastic recoiling in the sarcomere [7]. Titin also modulates the myosin-actin filament-mediated active force via non-cross bridge formation [10,11,12,13]. Structurally, titin is encoded by 363 exons translated into a protein with a molecular mass that ranges from 3800 to 4200 kDa, as dictated by its isoforms [2,9,14,15]. Titin is a modular protein interspersed with sections of tandem immunoglobulin (Ig)-like domains and fibronectin-type III (Fn III) domains [3]. Titin at the I-band region of the sarcomere is characterized by the presence of an N2B element, N2A insertion, and a stretch of amino acids rich in proline, glutamate, valine, and lysine (PEVK region) (Figure 1B) [16].

At the I-band, the N2A insertion of titin is regarded as a signaling hub due to its interactions with multiple binding partners involved in different cellular processes [18]. These binding partners include actin myofilament [19,20,21], muscle ankyrin repeat proteins (MARPs) [18,20,21,22], SET and MYND domain-containing protein 2 (SMYD2) [23,24], methylated HSP90 [23,25], proteases such as p94/calpain 3 and μ-calpain/calpain 1 [18,24], and a contraction modulator, calcium (Ca^2+^) [19,26]. The binding of N2A titin to these partners establishes connections between titin and biological cascades elicited by the binding partners. For example, p94 protease’s binding to N2A titin was indicated to forestall activation of the p94 protease, thus preventing its autolysis and the degradation of its sarcomeric targets [18,27]. MARPs are known as transcription modulators activated by stress in striated muscle [22]. Their binding interactions to N2A titin connect the N2A insertion as a stress-sensing or responsive area of titin [15]. Recently, SMYD2’s binding interaction to N2A was reported to help protect N2A or titin from proteolysis by proteases [24]. The N2A–actin-filament interaction mediated by CARP was found to be important to regulate the contractile force in the skeletal muscle [20,21]. These observations collectively highlight the significance of the N2A insertion for modulating titin’s activity and, by extension, sarcomere integrity, which brings a need for appraisal of the N2A domain in light of its interactions and sarcomere maintenance.

This review focuses on the N2A domain of titin, beginning by highlighting the presently available knowledge on its definition within titin and the sarcomere, its sub-domain composition, and structural characterization. Next, it will examine the molecular interactions that involve the N2A element under the purview of stress factors, chaperones/co-chaperones, sarcomeric proteins, and regulatory ligands. We will also appraise it in terms of its proteolysis by proteases in the context of the stress response or protein quality control. It will be concluded with remarks on the functional importance of the N2A domain’s interactions in striated muscle.

## 2. Structural Conformations of Titin’s N2A Domain

The N2A domain resides in the elastic I-band region of titin (Figure 1B) [7]. The expression of N2A is determined by titin splicing events that are linked to the cardiac location (e.g., left/right ventricle or atrium), developmental stage of striated muscle, species, and pathologic conditions [5,28,29]. The N2A isoform is primarily present in skeletal muscle, while the N2B isoform is found only in cardiac muscle [30]. When the N2A insertion is co-expressed with the N2B element in cardiac muscle, the isoform is referred to as N2BA (Figure 1B) [16]. N2A, N2B, and N2BA titin isoforms formed by alternative splicing have differential length and stiffness in striated muscle [7,31]. For example, titin isoforms predominantly expressing the N2B element (i.e., N2B titin) are shorter and stiff, while titin isoforms with an N2A insertion (i.e., N2A and N2BA titin) are longer and compliant [2,30].

The N2A segment is made up of four Ig domains (hereafter referred to as N2A sub-domains), annotated as Ig80, Ig81, Ig82, and Ig83, and an intervening 117-amino-acid sequence, known as a ‘unique insertion’ (uN2A), which is sandwiched between Ig80 and Ig81 (Figure 1C) [32]. The number of residues in the Ig sub-domains of N2A ranges from 87 to 140. From an investigation measuring sedimentation velocity, the molecular mass of N2A was calculated as 56.4 kDa, within the range of its theoretical mass of 59.5 kDa [14]. In the study, N2A was found to be mainly monomeric (85.3% monomers). It was also observed that N2A had the highest sedimentation coefficient (s_20,w_) amongst a panel of other constructs at the elastic region of titin, such as N2B and PEVK. The sedimentation coefficient shows the sedimentation velocity during centrifugation associated with the form and dimension of a molecule, and it has an inverse relationship with a frictional ratio (f/f_0_). The lowest frictional ratio associated with N2A depicts a relatively globular conformation of N2A, contrasting with N2B and PEVK, whose values indicated extended conformations. This result agrees with previous data that support the disordered nature of the N2B and PEVK segments [4,9,33].

The Ig81 domain was the first N2A sub-domain to be structurally characterized [34]. Due to the lack of structural information for other N2A sub-domains, the Ig81 structure was analyzed in the context of other reported titin Ig domains. It was observed that the Ig81 crystal structure features the canonical Ig-like fold of a two-layered β-sandwich that consists of the β-strands ABED and A’GFC (Figure 1D) [17]. Titin Ig-like domains have been classified, based on their sequence and structural attributes, as N-conserved or N-variable [34]. The N-conserved Ig domain is categorized based on a PP residue in the A-strand, a conserved PxP or PPh motif (where x is any residue and h is any hydrophobic residue) in the BC loop, and an NxxG definition in the FG loop [35]. The preserved motifs in the BC and FG loops of the N-conserved Ig subtype create loops longer than those in the N-variable counterpart. Conversely, the N-variable Ig subtype lacks these motifs with shorter BC and FG loop segments [35]. Interestingly, Ig81’s sequence does not conform to either canonical form of the N-conserved or N-variable subtypes, as it bears features from both classes [34]. For example, Ig81 lacks the PxP/PPh BC loop motif and NxxG in its FG loop. However, even without the conserved motif, its BC loop still appears longer than expected for an N-variable Ig subtype. These observations preclude the strict classification of Ig81 to either class. This non-stereotypical divergence of Ig81 has been suggested to be important for its interactions with binders, such as cardiac ankyrin repeat protein (CARP), a member of the stress-sensing transcription factors that belong to a family of muscle ankyrin repeat proteins (MARPs) [22,34].

In addition, the crystal structures of Ig81–Ig83 in tandem have been recently analyzed, showing their distinct structural variations [21]. Ig82’s conformation was depicted as an N-variable type because its BC and FG loops have a reduced length comparable to exemplary N-variable Ig domains, such as the I9–I11 region of titin [36]. On the other hand, Ig83 does not fit into either class, as observed in Ig81, because it has a shorter BC loop and a longer FG loop than expected. Similar observations were made by solution NMR analysis that corroborated the non-canonical structure of Ig83 [37]. Because the Ig80 structure has not been analyzed, it was built by structural modeling [38], which predicted Ig80 as a typical N-conserved Ig subtype. The apparent structural divergence seen in Ig domains of N2A is notable, especially with contrasting observations that titin’s other domains, such as the Ig65–Ig70 tandem, showed a high percentage of sequence similarity and structural homology [39]. Therefore, considering the predominantly homologous nature of titin’s Ig domains, the N2A’s structural variability may present its tendency to have unique protein–protein interactions.

In addition to the Ig sub-domains of the N2A region, the uN2A insertion sequence has been a focus of interest because various in vitro biophysical studies support that uN2A is unstructured [32]. By employing size exclusion chromatography with multi-angle laser light scattering (SEC-MALLS), the molecular mass of uN2A was calculated as 13.1 kDa, a value in close agreement with its theoretical mass of 13.3 kDa [34]. This value also confirmed the monomeric constitution of the uN2A domain. However, another study assessed the molecular mass of uN2A exclusively by using SEC [32], where uN2A migrated with a shorter retention time than its monomeric mass. Its molecular mass was predicted to be 45 kDa based on its retention time, which is about three times higher than its theoretical mass. This discrepancy observed from SEC analysis was also attested in an earlier study that found an early elution of uN2A [34]. Despite this discrepancy, the short retention time could indicate an oligomeric state of uN2A at its high concentration in vitro or an increased migration rate based on its conformation. When a glutaraldehyde cross-linking analysis was carried out on uN2A to evaluate its potential for oligomerization, the outcome was negative for oligomerization, suggesting that an irregular or extended conformation of uN2A may be a plausible reason for the short retention time [32]. The premise of an extended conformation of uN2A was validated by FRET analysis that determined the end-to-end distance of uN2A [32]. This evaluation found a longer length of uN2A at 5.2 nm, which is about 1.25 times longer than a typical Ig domain. Further corroboration of the extended or less compact uN2A conformation was observed in its 2D NMR analysis using NOESY and TOCSY, which showed that the NMR signals for uN2A were broad and did not correlate with expectations for a globular protein [34]. These outcomes indicate a less compact conformation of uN2A.

A protein’s amino acid sequence can reveal its intrinsic unstructured content [40,41]. To derive insights about the degree of structural disorder in uN2A from its sequences, in silico analyses were applied that showed varying outcomes [32,34]. Analysis with JPred and PONDR algorithms gave both unstructured and helical regions for a putative uN2A structure. Based on this observation, circular dichroism (CD) was applied on uN2A to derive an estimate of its secondary structure [42], and the CD spectrum depicted uN2A with an estimated helicity of 62% [34]. However, a similar CD study analyzed and calculated that uN2A has 15% helical content and 38% disordered structure content [32]. Such a discrepant and low helical content elicited whether there is a transitory conformation of uN2A between loose and compact helical conformations. To verify such a premise, uN2A was analyzed in the presence of different concentrations of 2,2,2-trifluoroethanol (TFE), a helical structure stabilizer, which showed a corresponding increase in its helicity to 31%. This observation suggests that uN2A may have regions with a transient character. It would be interesting to see whether such transitory conformation of uN2A plays a role with its interacting partners, such as CARP or calpain 3/p94.

Using a combination of NMR approaches and ab initio modeling, a recent report presented a plausible structure for the uN2A insertion [21]. Unlike the Ig sub-domains of N2A, the model derived for uN2A was not entirely resolved. The sequences flanking the N- and C-termini of uN2A were challenging to annotate due to overlapping signals in NMR, which primarily resulted from inherent flexibility or unstucturedness in the segments. Nevertheless, 46 amino acid sequences in the middle of uN2A were assigned as an α-helical conformation, which agrees with previous data about the helical nature of the uN2A insertion [32]. Extending from structural elucidations of individual sub-domains, structural determination of the composite N2A spanning Ig80–Ig83 (inclusive of uN2A) will help create a better picture of how the N2A domain influences titin’s structure and function.

## 3. N2A Domain—An Interaction Node in Titin

Titin is a multidomain protein, and these domains enable its involvement in protein–protein interactions [9]. Proteins with structural functions [16], contractile actin filament [43,44], chaperone, and co-chaperone activity [14,45], as well as transcription modulators [18,22], are amongst examples of titin’s binding proteins. Because these interactions may tune titin’s functions, the distinct domains of titin, such as N2A, that have multiple protein–protein interactions could be designated as binding ‘hubs’ [5]. The predisposition of the N2A domain for binding to other proteins may be associated with (i) its locus in titin and at the sarcomere, (ii) the elastic character of the I-band region of titin, and (iii) the inherent disposition of its Ig sub-domains [17,34]. The following sections will elaborate the binding partners of N2A within titin and their functional significance.

### 3.1. Ca^2+^ and F-Actin to Modulate Contraction

Titin is regarded as an entropic spring that produces passive force during sarcomere extension [46]. In addition to its elasticity, titin’s interaction with actin filament was noted early on as a contributor that causes a viscous force during stretching of cardiac myofibrils [46]. Among the I-band segment, titin’s PEVK region was shown to interact with actin filament, increasing titin’s stiffness during diastolic stretching and opposing the sarcomere shortening during active contraction [43,44]. Ca^2+^ bound with the calcium-binding protein S100A1 was found to abolish the titin–actin-filament interaction, which could free the actin filament from titin before actin–myosin active contraction. Despite these analyses, recent data support a negligible increase in titin-based stiffness and force during active eccentric contractions of cardiac muscle [47].

More recently, the titin–actin-filament interaction has been a focus of interest in the eccentric contractions of skeletal muscle. It has been noted that actin–myosin cross-bridge (XB) formation suffices for force generation in concentric and isometric contractions [10]. On the other hand, XB does not explain the increased force following active eccentric contraction [13], nor the force enhancement and force depression in isometric contraction [12]. Therefore, non-cross-bridge (non-XB) formation was suggested to explain the additional force, defined as residual force enhancement (rFE), observed in eccentric contractions [13]. In this regard, titin–actin-filament interaction, especially via titin-PEVK binding to actin, in the skeletal muscle may account for the additional forces generated beyond myosin–actin interactions [46,48]. However, in addition to titin-PEVK regions, titin’s N2A domain has recently emerged as an important interaction node between titin and actin filament in skeletal muscle under the control of Ca^2+^ or CARP [19,20,21].

**Figure 2:**
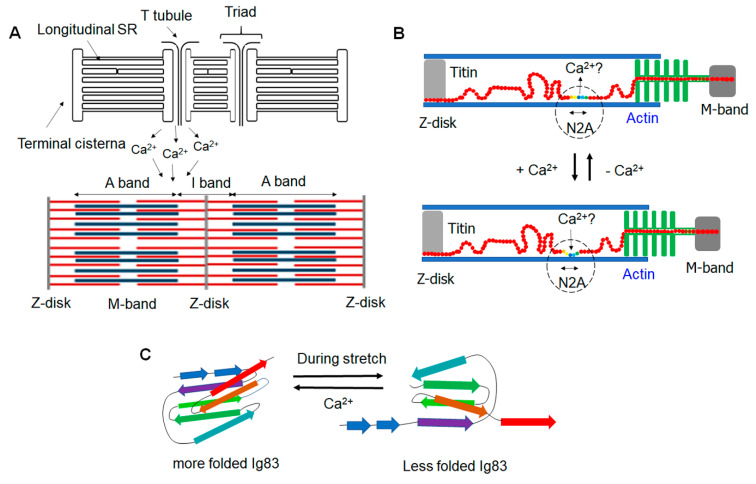
Calcium ion’s interactions with the N2A domain of titin during contractions and stretching. (**A**) Alignment of the sarcomere and sarcoplasmic reticulum (SR). The SR stores and releases calcium ions toward the sarcomere. The triad structure made of the SR and T-tubules overlaps with the sarcomere’s A-I junction close to the N2A region of titin. (**B**) The potential role of calcium ion in the N2A–actin-filament interactions. The influx of Ca^2+^ may enhance the N2A’s binding with actin filaments, which needs additional investigation due to conflicting data. (**C**) The role of calcium ion in stabilizing the N2A’s sub-domain. The Ig83 domain is stabilized and keeps a more folded structure in the presence of calcium ion, whereas it may be subjected to unfolding during stretches of titin. (**A**) was adapted and redrawn from Rossi et al.’s publication [51].

The Ca^2+^-sensitive actin filament and its regulatory proteins (troponin and tropomyosin) are aligned with titin’s elastic domains that bear the N2A locus [49]. Additionally, the A-I band junction of the sarcomere overlaps with a triad structure formed by the sarcoplasmic reticulum (SR) and adjacent T-tubules that store Ca^2+^, placing the N2A domain in the proximity of the Ca^2+^ repository (Figure 2A) [1,50]. These factors instinctively place the N2A domain under the influence of Ca^2+^, justifying its tendency to interact with the regulatory Ca^2+^.

Due to the importance of Ca^2+^ in regulating contractile activity, it was hypothesized that Ca^2+^ is important for modulating the binding affinity of actin filament for titin during contraction, specifically via the N2A domain [19]. Dutta et al. have shown that a recombinant N2A construct (full-length N2A) binds F-actin, with the affinity influenced by Ca^2+^ [19]. A co-sedimentation assay of N2A and F-actin showed that the amount of N2A bound to F-actin increased progressively in the presence of Ca^2+^, with a stronger binding interaction between N2A and F-actin at high (pCa = 4) versus low Ca^2+^ (pCa = 10) concentrations. Furthermore, single-molecule force spectroscopy (SMFS) was employed to analyze the stability of their interactions when Ca^2+^ was present. It was found that a higher pulling force was required to dissociate the N2A–actin complex in the presence of Ca^2+^ than in its absence [19]. Direct force spectroscopy (DFS) analysis indicated that the k_off_ value between N2A–actin interactions in the presence of Ca^2+^ was three times higher than that recorded without Ca^2+^. These findings suggest a potential role of Ca^2^ in mediating N2A–actin interaction during contraction (Figure 2B).

However, the N2A–F-actin interaction under the influence of Ca^2+^ [19], which falls in line with the three-filament postulate [10], is inconclusive based on conflicting reports [20,21]. Zhou et al. evaluated the interaction of actin filaments with two N2A constructs, uN2A-Ig81 and Ig81–Ig83, which failed to recapitulate actin’s binding with such N2A constructs [38]. In vitro co-sedimentation assays of uN2A-Ig81 and Ig81–Ig83 constructs with actin filaments did not show complex formation in the presence or absence of Ca^2+^. Furthermore, C2C12 myoblast and differentiated cells transfected with fluorescent protein-labeled Ig80-uN2A-Ig81 or Ig81–Ig83 showed no co-localization with actin [38]. On the other hand, a recent report supports that the N2A sub-domain construct Ig80-uN2A-Ig81 binds to F-actin, whereas the deletion construct Ig80-Ig81 (missing uN2A) does not show such binding [20]. Although this report is consistent with the study of Dutta et al., it was observed that the addition of EGTA (a Ca^2+^ chelator) to the binding cocktail was rather beneficial for the binding between Ig80-uN2A-Ig81 and F-actin. In the face of these conflicting outcomes, more concordant data were reported by two independent groups, showing that the interaction between titin’s N2A domain and actin filament is mediated or strengthened by the interaction with CARP (discussed more later).

### 3.2. Ca^2+^ to Regulate the Stability of N2A’s Ig Domains

The N2A domain is positioned between proximal Ig domains and PEVK in the elastic I-band [32]. At the I-band, N2A has been presumed to act as a ‘buffer’ that prevents a sarcomere from being overstretched under high forces, especially by unfolding its immunoglobulin sub-domains [26]. Because of the potential folding and unfolding nature of Ig sub-domains, the inherent variation in the stability of individual N2A sub-domains has been investigated.

Tryptophan fluorescence and center of mass (CoM) calculations were applied to monitor the unfolding rate of recombinant constructs of N2A sub-domains (individual Ig81, Ig82, and Ig83 constructs) in the presence of varying concentrations of urea [26]. It was observed that Ig81 was the most stable, while Ig83 was the least stable. The midpoint unfolding values for Ig81 and Ig82 were similar, suggesting their comparable stability. However, Ig82 showed a broad transition in unfolding, which was interpreted as less cooperative unfolding than Ig81. Since Ig81 showed the highest chemical stability and Ig83 had the lowest, it was concluded that Ig81 would equilibrate towards the folded state and Ig83 towards the unfolded state. Notably, the sequence analyses showed a 24% similarity between Ig81 and Ig83. This low sequence similarity is significant because immunoglobulins in tandem with a lower sequence similarity tend to be more stable during stretching by being refolded adequately than those in tandem with high similarities >40% [52]. It was concluded that this intrinsic property with low sequence similarity could be a feature of N2A sub-domains to avoid misfolding when unfolded.

Considering the supposed role of Ca^2+^ for the interaction between N2A and F-actin [19], the putative implication of Ca^2+^ in the stability of N2A sub-domains was envisioned. In addition, because Ca^2+^ plays a role in tuning the stability of Ig domains [53], it was suspected that the stability of individual Ig sub-domains of N2A might be influenced by Ca^2+^ availability, which could serve as a potential mechanism that preserves N2A from overstretching [26].

An investigation of individual N2A sub-domains using their intrinsic fluorescence revealed that Ig83’s stability was increased in the presence of physiological levels of Ca^2+^ at the sarcomere (with a 30% change recorded for the m-point). In contrast, the stability of Ig81 and Ig82 remained unchanged [26]. These observations indicated that the presence of Ca^2+^ could lead to a more folded state of Ig83 by stabilizing its structure (Figure 2C). Interestingly, Ig83 is abundant in glutamic acid residue that has a high affinity for Ca^2+^, which could be a molecular basis for Ig83’s interaction with Ca^2+^. However, Ig82 also has many glutamic acid residues, and its stability was not impacted by elevated Ca^2+^ levels [26]. Therefore, it was thought that Ig82 might not be responsive to Ca^2+^, or binding to Ca^2+^ may elicit other unidentified effects. Despite these analyses, additional experiments are necessary to investigate whether Ig83’s increased stability by Ca^2+^ influences titin-based stiffness or force generation.

Other studies to delineate Ca^2+^ and N2A binding have suffered divergent outcomes. A bioinformatics analysis of Ig81–Ig83 did not find the electrostatic surface potential or any hotspots that can coordinate a divalent cation, such as Ca^2+^ [38]. Furthermore, prediction algorithms that search for metal ion binding sites in the structure did not identify Ca^2+^-binding sites [38]. These results suggest an unlikelihood or non-specificity of Ca^2+^ interaction with Ig83. However, in contrast to these bioinformatic analyses, biophysical analyses of the Ig83 sub-domain yielded positive outcomes for its interactions with Ca^2+^ [37]. NMR analyses showed increased chemical shift perturbations (CSPs) of Ig83 residues in the presence of Ca^2+^. It was found that three negatively charged residues (D36, D37, and E86) themselves were in proximity, implying their potential coordination to Ca^2+^ [37]. Since D36, D37, and E86 are within the BC and FG loops, it is suspected that the unique attributes of these loops in Ig83 may allow for their spatial coordination with Ca^2+^ [38]. Additionally, Ca^2+^ site mutants (D36N, D37N, and E86Q) were used to investigate the role of three residues in binding to Ca^2+^. Structural stabilization by Ca^2+^ was not significantly seen with either single mutants or a triple mutant of Ig83, as opposed to wild-type Ig83 [26], indicating that D36, D37, and E86 in Ig83 may constitute a site that coordinates with Ca^2+^ [37]. While this biophysical study focused on Ig83, it may be helpful to assess other N2A sub-domains in similar analyses to characterize their responses to Ca^2+^. In addition, the same examination with an N2A full-length construct may provide information on how end-to-end joining between sub-domains impacts N2A’s conformation in the presence of Ca^2+^.

### 3.3. HSP90 and SMYD2

The complexity of the sarcomere and its dynamic nature inevitably place a strain on its components [54]. Titin is not occluded from this imposition, as its integral nature makes it a direct target of a gamut of factors in the sarcoplasm [2]. The Ig domains in titin’s elastic region may respond to stretching forces by unfolding and refolding them [55]. Their unfolding can expose the normally buried hydrophobic amino acids [14], thus presenting them to potential biochemical perturbations at the sarcomere during excitation–contraction coupling. Among possible events, Ig domain unfolding poses an opportunity for aggregation of the unprotected hydrophobic patches [14], which can elicit binding interactions with heat shock protein (HSP) chaperones.

The heat shock protein (HSP) family is the prevalent biological ‘guardian’ of proteins. Its expression is triggered by aberrant cellular conditions to help protein refolding and avoid protein aggregation or degradation [45,56]. Its ubiquitous member, HSP90, has been reported as a chaperone for myosin in the sarcomere [45]. HSP90 has not been reported to target titin directly. However, its methylated form, methyl-HSP90, has been shown to play the role of a chaperone with protein lysine methyltransferase, SMYD2, in both cardiac and skeletal muscle cells [23,25]. SMYD2 has been recognized for transcriptional regulation by histone methylation [56], but its substantial cytoplasmic expression in skeletal muscle cells has suggested its roles beyond transcriptional regulation [23]. Overexpression of SMYD2 also coincides with a high level of methyl-HSP90 in cell extracts upon addition of S-adenosyl-methionine (SAM) [23]. Interestingly, SMYD2 did not methylate HSP70, a member of the HSP family, indicating SMYD2’s preference for HSP90. A mass spectrometry analysis showed that HSP90 was mono-methylated by SMYD2 at lysine 616 (K616) [23].

**Figure 3:**
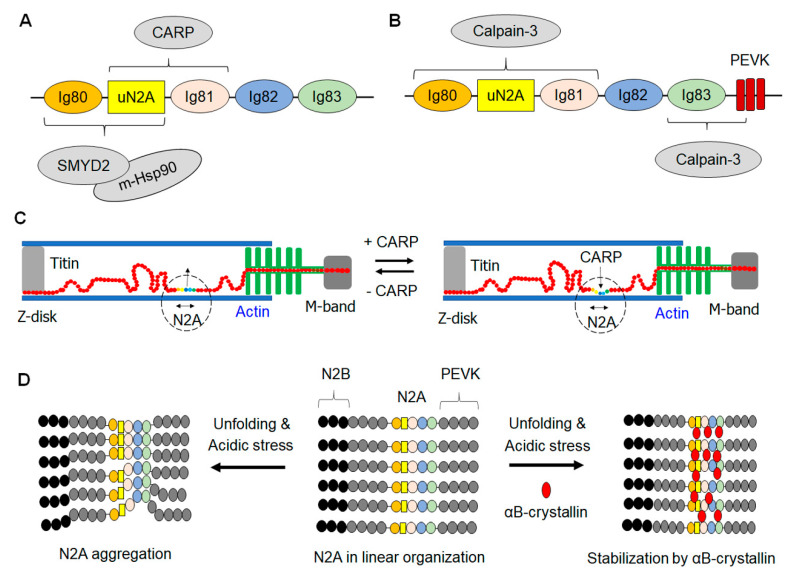
N2A’s interacting partners and potential roles of N2A–protein interactions. (**A**) N2A’s interactions with SMYD2, methyl-Hsp90, and CARP. SMYD2–methyl-Hsp90 and CARP play chaperone roles in protecting or stabilizing their interacting N2A sub-domains against protease activity or unfolding events. (**B**) N2A’s interactions with calpain-3. Calpain-3 binds at least two regions that encompass the N2A sub-domains. N2A’s interaction with calpain-3 makes the calpain-3 inactive, thus regulating the calpain-3 protease activity. (**C**) N2A–F-actin complex formation in the presence of CARP. CARP locks titin’s N2A domain to the actin filament, thus increasing titin’s passive force or stiffness during skeletal myofibril stretch. (**D**) N2A’s interactions with αB-crystallin. N2A is prone to aggregation in an acidic environment upon unfolding that exposes its buried hydrophobic cores. αB-crystallin binds and stabilizes the N2A region to prevent its aggregation. (**D**) was adapted and redrawn from Kotter et al.’s publication [14].

The tripartite connection of methyl-HSP90, SMYD2, and N2A (Figure 3A) was recognized by co-localization of GFP-tagged SMYD2 around α-actinin within Z-disks in primary chick skeletal myocytes [23]. In addition, co-localization of methyl-HSP90 and GFP-SMYD2 was also observed, indicating the potential complex formation of SMYD2 and methyl-HSP90 in the I-band region of the sarcomere. Furthermore, it was observed that the distance between SMYD2 and α-actinin at the Z-disk in stretched myofibrils was the same as that between the N2A region and the Z-disk, which suggests that SMYD2 may be co-localized with the N2A domain of titin. The subsequent yeast two-hybrid (YTH) screens and GST pull-down assays substantiated their findings on the SMYD2 and N2A interaction. They also indicated that the sequences coded by titin’s exon 104 (a part of uN2A) are responsible for binding to SMYD2 (Figure 3A). Intriguingly, N2A was devoid of any methylation by SMYD2 [23], suggesting that N2A is not directly regulated by SMYD2 enzymatic activity. In a similar study, cardiomyocytes were also probed with SMYD2 and methyl-HSP90 antibodies, which confirmed their localizations along α-actinin in the Z-disk region [25]. It was also notable that the methyl-HSP90 labeling aligned with the N2A region, whereas unmethylated HSP90 was dispersed in the cytoplasm [25]. These findings implicate the ternary complex formation by titin N2A, SMYD2, and methyl-HSP90 at the I-band.

Since methyl-HSP90 co-localized with SMYD2 at the N2A locus, this observation raised the next question about the coordination of their biochemical interactions. C2C12 myoblasts were transfected with SMYD2 or N2A constructs. The subsequent pull-down confirmed that methyl-HSP90 forms a complex with SMYD2 [23]. Similarly, N2A was shown to pull down the SMYD2–methyl-HSP90 complex. Notably, in the absence of SMYD2, the interaction of HSP90 with N2A was lost, indicating that HSP90 methylation by SMYD2 is necessary for complex formation between methyl-HSP90 and N2A. The importance of this ternary complex in the sarcomere was demonstrated by introducing an inactive mutant of SMYD2 to C2C12 myoblasts, which caused a significant decrease in N2A expression levels [23]. Importantly, this decrease in N2A expression correlated with a loss of titin’s structural integrity, as monitored by titin N2A immunoblotting [23]. In addition, the knockdown of SMYD2 by its siRNA resulted in phenotypes with reduced immunostaining of the N2A region [23], suggesting a decrease in the N2A titin’s expression without SMYD2. Although no report has been made investigating the role of the SMYD2–methyl-HSP90 complex in the stability or unfolding dynamics of N2A (nor its sub-domains), these observations may indicate a function of the tricomplex on the stability of titin or N2A. An interesting observation of the interaction between N2A and SMYD2 is that SMYD2’s oxidation influences its binding to N2A. SMYD2 glutathionylation at its Cys13 residue has been shown to impact its binding to N2A [24]. Using GST pull-down, it was observed that glutathionylated SMYD2 had a reduced binding with GST-N2A, and glutathionylated SMYD2 lost its co-localization with titin in neonatal cardiomyocytes. These findings have been further extended to suggest the role of SMYD2 in protecting N2A or titin from proteolytic degradation [24].

### 3.4. Other Small HSPs

In addition to the classical HSP chaperones, small HSPs (sHSP), such as αB-crystallin and HSP27, mediate protein refolding [45]. αB-crystallin was found to be predominant around Z-disk and I-band regions, especially with its high expression in cardiomyocytes, where it was suggested to help with titin folding [45]. GST pull-down assays showed that HSP27 and αB-crystallin interact with the N2B element and N2A in the elastic region of titin [14]. Although N2A has been reported as a monomer in vitro [14], the in silico protein aggregation predictor TANGO predicted a high β-aggregation score (>60%) for N2A, which indicates its high probability of unfolding-induced aggregation [14]. Based on the in silico β-aggregation score of N2A, denaturation analyses were applied to evaluate N2A’s unfolding and propensity for aggregation [14]. N2A did not aggregate in denaturation studies when denaturation was induced with 8 M urea at a neutral pH 7.4. However, it aggregated at acidic pH in the presence of 8 M urea. Interestingly, it was observed that the addition of αB-crystallin reduced the level of N2A aggregation, supporting that αB-crystallin protects titin from its aggregation and stiffening (Figure 3D). It is also notable that the role of αB-crystallin was specific because HSP27 did not prevent N2A aggregation.

Despite the accumulating and supportive data, the studies on chaperones’ role in unfolding and refolding Ig domains or protecting Ig domains from potential aggregation are relatively limited. Additional experiments will help understand the chaperones’ definitive role in titin’s integrity.

### 3.5. CARP

Titin’s elastic region is reputed as a mechanosensor [7], and N2A in the elastic segment is suggested to be impacted by mechanical stress that results from contractile activity [54], making N2A a putative target of stress factors [22]. MARP proteins (MARP1/ankrd1/CARP, MARP2/ankrd2/Arpp, and MARP3/DARP) are transcription factors expressed in striated muscle and are known for their upregulation during stress [54]. Among the MARPs, CARP was found to bind to N2A (Figure 3A), which was revealed from a YTH screen using N2A as bait against a library of about a million clones derived from a human skeletal muscle cDNA library [22]. As a corollary, based on the homology of CARP with other members of the MARP family, the possibility of N2A’s interaction with DARP was surmised, which was also verified by YTH screens. Subsequently, additional YTH screens with deletion constructs of N2A sub-domains delineated that uN2A insertion and its flanking domains (Ig80-uN2A-Ig81) of N2A bind to CARP and DARP. Intracellularly, CARP’s affinity for the N2A titin domain was supported by its co-localization within the N2A region of titin in cardiomyocytes [22].

Insights to the N2A–CARP interaction led to additional investigations to verify the specific interacting sub-domains within N2A [34]. CARP^106−319^–N2A binding was characterized using truncated sub-domain constructs of N2A, and the study showed that the uN2A–Ig81 construct bound CARP^106−319^ (Figure 3A). Neither uN2A nor Ig81 alone showed significant binding with CARP^106−319^, emphasizing the requirement of both tandem domains for binding [34]. Although dimerization has been observed with CARP, the SEC-MALLS analysis showed that CARP^106−319^-uN2A-Ig81 forms a monomeric complex. Hydrogen/deuterium exchange mass spectrometry (HDX-MS) was recently used to determine crucial interacting residues at the interface between CARP^106−319^ and uN2A-Ig81. The analysis delineated that the interacting interface consisted of (i) the central helix region of uN2A, (ii) the intervening linker between uN2A’s C-terminus and Ig81, and (iii) Ig81’s BC loop [21], showing broad binding motifs within uN2A-Ig81. These findings highlight the distinct features of N2A’s Ig sub-domains for mediating its interactions with binding partners.

CARP is a co-transcriptional regulator whose expression is high at the embryonic stage but low in the adult heart [57,58,59]. Mechanistically, it suppresses the transcription of genes involved in adult cardiac maturation while inducing fetal genes. CARP has a short lifetime due to ubiquitination-mediated proteasomal degradation [60]. However, its stabilization has been observed under mechanical or oxidative stress [59]. Upregulation or stabilization of CARP under stress conditions or during heart failure [61] is considered the response for the remodeling of cardiomyocytes by inducing fetal genes that would comply or cope better with the stress condition [57]. In addition to transcriptional regulation, CARP was suggested to localize at the sarcomere, especially binding to the N2A region. Importantly, the functional effect of CARP for interaction with N2A was investigated by testing the unfolding dynamics of N2A constructs in the presence of CARP [62]. In this study, the binding of CARP to the N2A constructs resulted in a resistance to unfolding, with a higher unfolding force (F_unfold_) necessary to unfold the domains, suggesting that CARP plays a role as a molecular chaperone in stabilizing N2A that is prone to unfolding aberrantly or aggregating under mechanical stress. It is interesting to note that a serine residue in uN2A-Ig81 was phosphorylated by protein kinase A (PKA). However, the phosphorylation did not impact the mechanical property or unfolding of uN2A-Ig81 [62]. Instead, it was found that CARP blocked the phosphorylation of uN2A, possibly due to CARP–uN2A interactions that physically compromise PKA binding to uN2A.

Recently, two independent studies reported an important role of CARP in modulating the sarcomere’s mechanical force. It was found that CARP mediates N2A’s binding to F-actin by forming the CARP–N2A–F-actin complex (Figure 3C) [20,21]. N2A–F-actin binding was not apparent in the presence or absence of Ca^2+^ but was signified in the presence of CARP [21]. Importantly, CARP’s interaction at the N2A–actin locus enhances the passive force of the sarcomere by locking N2A of titin to actin filament and subsequently allowing the elongation of the PEVK region [20]. In the investigation, myofibrils derived from the skeletal muscle of triple MARP knockout mice showed an enhanced passive force upon addition of exogenous CARP. Additionally, an increased passive force was found in myofibrils derived from the skeletal muscle of the diaphragm of acutely ill patients undergoing mechanical ventilation, which is associated with elevated CARP levels [20]. Elevated passive tension was also seen in myofibrils from skinned psoas mouse skeletal muscles infused with CARP^106−319^ [21]. These findings open up a new perspective that CARP regulates sarcomere mechanics by cross-bridging titin’s N2A domain to actin filaments.

It is important to indicate that Ankrd2/Arpp is a counterpart of CARP in skeletal muscle. Ankrd2/Arpp is abundant in human skeletal muscle, approximately 30-fold and 150-fold more so than DARP and CARP, respectively [63]. Additionally, Ankrd2/Arpp is induced after stretching skeletal muscle [22]. Therefore, Ankrd2/Arpp requires mechanistic evaluations of its interaction with N2A titin. Indeed, peptides from Ankrd2/Arpp’s second ankyrin repeat were shown to interact with N2A, supporting the Ankrd2/Arpp interaction with N2A [22]. As Ankrd2/Arpp is a part of the mechanosensory complex in the I-band involving N2A titin and calpain 3 [18,63], Ankrd2–N2A interaction may be important for the regulation and maintenance of contractile activity in the skeletal muscle.

### 3.6. Calpain

Calpain-3 (p94) belongs to the calpain family of Ca^2+^-dependent proteases ubiquitous in the body [64]. Calpain-3 is predominantly expressed in skeletal muscle [65], where titin was found to be a binding partner and a target of calpain-3 at the sarcomere. Interestingly, calpain-3 has the inherent property of autolysis after its activation [27], thus regulating its own protease activity in the muscle cell. However, when calpain-3 binds to titin, it remains in a dormant form, suggesting that its dissociation from titin would restore its protease activity [18]. The significance of calpain-3’s interaction with titin was investigated in mice with muscular dystrophy with myositis (mdm) that showed an abrogation of calpain-3’s association with titin in addition to severe pathological phenotypes [66].

Mdm arises from a deletion mutation of 83 amino acids in the Ig83/PEVK junction of titin that includes a part of N2A sub-domains. The deletion mutation was found to cause a loss of calpain’s binding to titin [66,67]. The absence of the calpain-3 binding site in mdm was surmised to induce calpain-3’s activation with subsequent digestion of its targets and severe phenotypes of mdm, such as extreme kyphosis and small body sizes (see Nishikawa et al., 2020, for a review on the influence of the mdm mutation) [67]. However, although the Ig83/PEVK junction of titin is implicated as calpain-3’s binding site, an extensive analysis by Hayashi et al. revealed the complex nature of calpain-3’s interactions with the N2A region [18]. Investigation with various constructs spanning N2A-PEVK segments showed that the Ig83-PEVK region serves as the primary binding site, and an additional binding site was observed in the Ig80-uN2A-Ig81 area (N2A sub-domains) (Figure 3B) [18]. Notably, an Ig82-Ig83-PEVK construct lacking the additional calpain-3 binding site abrogated its association with calpain-3. This observation signifies the importance of both N2A sub-domains and the Ig83-PEVK region for attaining calpain-3 binding to titin.

While mdm studies have highlighted the importance of N2A-PEVK for binding and regulating calpain-3, it remains unclear how calpain-3 relieves its binding interaction from N2A. It is thought that multiple forms of stress may contribute to the dissociation and activation of calpain-3 from titin [18]. In addition, an additional study demonstrated that overexpression of calpain-3 does not recapitulate the mdm phenotype, thus suggesting a complex nature of the deletion mutation of titin in mdm beyond the calpain-3 activity [66].

## 4. N2A Domain’s Susceptibility to Proteolysis

Striated muscle is an intricate tissue with an assembly of myofibrillar, sarcoplasmic, and stroma proteins [27,68]. These proteins form a dynamic system at the sarcomere and are physiologically maintained by the sarcomere’s protein quality control systems [69], such as the ubiquitin–proteasomal system (UPS) and the autophagy/lysosome system [68,70]. Notably, beyond the UPS and autophagy/lysosome systems, there are many proteases that have both physiological and pathophysiological effects on sarcomere proteins [27]. Examples include calpains, Ca^2+^-dependent proteases, and matrix metalloproteinases (MMPs), which are known for their roles in extracellular matrix remodeling [71,72]. These proteases have been implicated in aberrant muscle conditions, such as atrophy, that are characterized by an imbalance of sarcomere protein synthesis and breakdown [27].

The N2A domain of titin was considered susceptible to proteases because of its locus at the stress-sensitive and elastic I-band segment of titin [73]. N2A’s association with chaperones, such as αB-crystallin and HSP90, under stress conditions indicates a potential local alteration (or loss) of its conformational stability, which also hints that N2A may potentially be targeted by proteases [74]. This notion was further supported by observing a recombinant N2A construct that aggregated in acidic buffer, unlike other distinct regions of titin with a disordered nature, such as the N2B element and PEVK stretch [14,75]. Therefore, the N2A domain was suggested as an important node in titin that may need to be protected from proteases to stall the destructive degradation of titin [18].

### 4.1. Proteolysis by Calpains

Early connection of the N2A region and proteases was seen in post-mortem tissue by degradation of titin and other muscle proteins [76]. Subsequently, calpains were shown to be involved in the degradation of proteins in post-mortem tissue, ischemic injury [77], and muscle atrophy [27]. Among the calpain family, calpain-3 and calpain-1 have been implicated in the breakdown of titin, and the N2A region of titin was indicated as a node of degradation by both calpains [18,24]. Therefore, the N2A region of titin is not only implicated in binding and regulating the calpain-3 activity but is also susceptible to degradation by the calpain family.

Calpain-3’s putative interaction area on titin was initially observed by its antibody labeling at Z-line, N2A, and M-line segments in mouse skeletal muscle cells [78]. Subsequently, based on prior reports about calpain-3 interaction with titin in the N2A-PEVK region in an mdm model [18], the expression level or stability of the N2A-PEVK construct was examined upon expression of calpain-3. When N2A-PEVK and calpain-3 wild type (WT) were co-expressed in COS7 and Sf-9 cells, a decrease in the levels of N2A-PEVK was observed. In a supporting analysis, a similar experiment with an inactive calpain-3 mutant (calpain 3:C129S) resulted in a higher residual level of N2A-PEVK, indicating that the N2A-PEVK intersection could be a direct substrate of calpain-3. Because calpain-3 remains dormant or inactive upon binding to the junction within N2A and PEVK, these data imply that the dissociation of calpain-3 from the N2A-PEVK of titin is necessary for the activation of calpain-3 and the degradation of titin at the N2A-PEVK region.

In addition to calpain-3, titin’s disappearance amongst a panel of sarcomere proteins was observed when human left ventricle cardiac tissue was treated with calpain-1 [72], suggesting titin’s degradation by calpain-1. Interestingly, an immunostaining experiment of bovine myofiber with calpain-1 antibody showed that calpain-1 was interspersed between the Z-disk and the mid-section of the I-band region [76], supporting its localization around the N2A region or at the I-band. Additional analyses have shown that the N2BA titin isoform containing the N2A domain was degraded into multiple fragments by calpain-1 [72]. Calpain-1 was also demonstrated to digest recombinant N2A constructs [76], supporting that N2A is one of the titin sub-domains susceptible to degradation by calpain-1. As calpain-1 does not suffer from autolysis, unlike calpain-3, it was possible to investigate its direct substrates or degraded sequences in biochemical studies [24]. The sequences within N2A degraded by calpain-1 have been analyzed and mapped to two sites at the N- and C-terminal borders of N2A’s unique insertion (uN2A) [18].

### 4.2. Proteolysis by MMP-2

MMP-2 is one of the major proteases that has significance in striated muscle [79]. It belongs to a family of proteases originally recognized for their roles in remodeling the extracellular matrix [71]. Although MMP-2 has been noted for its extracellular roles, its intracellular activities have also been identified [80], connected with its intracellular expression [81]. Its intracellular expression is attributed to the loss of a signal sequence that leads to its inadequate export [81]. Alternatively, induction of a cytoplasmic isoform has been found under oxidative stress [80,82]. Consequently, MMP-2 has been shown to degrade intracellular proteins [83,84] in normal and disease conditions [85]. A full-length MMP-2 construct is inactive but activated upon proteolytic cleavage or post-translational modifications [85]. Oxidative stress has been identified to activate its activity [85], as observed in heart muscle [86].

In addition to calpains, titin is also degraded by MMP-2 [85], which may not be unusual as both proteases are known to be activated by oxidative stress [86]. The degradation of titin by MMP-2 has been observed in rat cardiac tissue following ischemic reperfusion [85]. In this evaluation, after 10 min of reperfusion, the heart left ventricle showed co-localization of MMP-2 and titin at the Z-disk. MMP-2 staining was also seen at the M-line, albeit with lower intensity. The subsequent in vitro analysis showed that MMP-2 digests titin in a concentration-dependent manner, which was suppressed in the presence of its inhibitors, such as GM-6001 and ONO-4817. The N2A domain was then suggested as one of the titin sub-domains digested by MMP-2 [24]. Recombinant N2A treated with MMP-2 was fragmented by MMP-2 in a concentration-dependent manner, which was inhibited in the presence of SMYD2 that binds to the N2A domain. These data support the N2A domain as an important node regulated by a balance between MMP-2 protease and the SMYD2-HSP90 chaperone.

Degradation of the N2A domain by both calpain-1 and MMP-2 correlated with the observation that there are overlapping substrates between MMP-2 and calpains in cells [80]. It is also noteworthy that both calpain-1 and MMP-2 are inducible by oxidative stress and degrade the N2A domain or an elastic region of titin in the I-band [80], suggesting that it may be important to prevent or mitigate proteolysis of the N2A domain or elastic region of titin.

## 5. Conclusions

Investigations on N2A titin have been informative about its role in preserving the integrity of titin and sarcomeres [24]. As a signaling node, N2A’s interaction with MARPs was speculated as a link between stress-sensing in the muscle cells and gene expression [15]. It is notable that MARPs were shown to be dispensable in heart development and hypertrophic responses [59]. However, recent data highlight the effect of CARP in aiding N2A–actin complex formation and regulating force generation [38]. In addition, the CARP–N2A interaction could be examined for CARP’s role in preventing the proteolytic susceptibility of N2A during oxidative stress in cardiomyocytes.

Although the N2A domain has been identified to interact with partners individually [67], multiple binding interactions [18] converge on the proximal sites within N2A, which creates an intricate network of interactions or signalosomes. For example, in the complex of N2A–SMYD2–methyl-HSP90 [25], the N2A domain recruits SMYD2 to interact with methyl-HSP90. Such a complex of SMYD2–methyl-HSP90 confers protection for the N2A region from degradation that may adversely affect myofibril integrity. Similarly, in the case of calpain-3–N2A interaction, the N2A-PEVK domain has multiple binding sites for calpain-3 [18]. Calpain-3’s binding site within Ig80-uN2A-Ig81 overlaps with CARP’s interaction site within titin [18]. Therefore, the coordination of CARP and calpain-3 within each other’s vicinity is posited to protect the proteolytic sites within N2A titin from degradation under oxidative stress [18].

The N2A locus of titin is documented as an important region of titin for coordinating signals in the sarcomere and relaying them to downstream effects. It will be important to continue revealing the molecular basis for the N2A structure, its interactions with binding partners, and the physiological and pathological significance of the N2A domain in titin.

## Data Availability

Not applicable.

## Abbreviations

FRET: Fluorescence resonance energy transfer; NOESY: Nuclear Overhauser effect spectroscopy; TOCSY: Total correlation spectroscopy; NMR: Nuclear magnetic resonance spectroscopy; PONDR: Predictor of natural disordered regions; H/D: Hydrogen/Deuterium; EGTA: Ethylene glycol-bis (β-aminoethyl ether)-N,N,N′,N′-tetraacetic acid.
